# The Effect of the Addition of Dietary Fibers from Apple and Oat on the Rheological and Textural Properties of Waxy Potato Starch

**DOI:** 10.3390/polym12020321

**Published:** 2020-02-04

**Authors:** Greta Adamczyk, Magdalena Krystyjan, Grażyna Jaworska

**Affiliations:** 1 Institute of Food Technology and Nutrition, Department of Food Technology and Human Nutrition, University of Rzeszow, Zelwerowicza Street 4, 35-601 Rzeszow, Poland; rrgjawor@cyf-kr.edu.pl; 2 Department of Carbohydrates Technology, Faculty of Food Technology, University of Agriculture in Krakow, Balicka Street 122, 30-149 Krakow, Poland; m.krystyjan@gmail.com

**Keywords:** starch, plant fiber, pasting properties, flow curves, anti-thixotropy and thixotropy

## Abstract

The aim of this paper was to investigate the influence of dietary fibers from oat (OF) and apple (AF) (concentration 0.2%) on the pasting properties, rheological (including thixotropic and anti-thixotropic) and textural properties of 3% and 4% (*w*/*w*) waxy potato starch pastes. The samples were characterized by their pasting characteristics, the hysteresis loop test, and textural properties measured during storage. It was found that the breakdown viscosity values of the blends, including oat fibers, were lower than those of the others (waxy potato starch (WPS), WPS-AF), which suggests that these samples would have higher resistance to retrogradation and therefore would form a more stable paste. The pattern of flow curves showed that the investigated waxy potato starch and starch-fiber pastes were non-Newtonian fluids, thinned by shear. Areas of the hysteresis loops indicated that pastes with fibers had anti-thixotropic or mixed thixotropic/anti-thixotropic character. The greatest areas of the anti-thixotropy hysteresis loops were characteristic for WPS, while its mixtures with AF and OF caused a decrease in the value of these areas. It can indicate that starch-fiber blends were more stable during shearing. Fiber-type and starch concentration strongly affected the textural parameters of the starch-fiber gels.

## 1. Introduction

Growing consumer awareness about healthy food forces producers to design convenient, functional, and safe food rich in vitamins, dietary fiber, and antioxidants [1,2]. Dietary fiber (DF) is an important component of the human diet with high physiological importance due to its resistance to digestion and absorption in the small intestine. Carbohydrate-based moiety of DF includes cellulose, β-glucans, hemicelluloses (arabinoxylans and arabinogalactans), pectins, gums, and mucilages [3,4].

DF has been the subject of intensive and comprehensive research in recent years. The reason for using dietary fiber is the constantly growing interest in healthy food, and thus bioactive substances found in plants, as well as ingredients and raw materials with functional properties, important in the technological process [5].

Dietary fiber plays an important role in the design of functional foods. Total dietary fiber (TDF) includes two fractions: soluble (SDF) and insoluble (IDF). Fibers of cereal preparations (wheat, oats) have a high IDF content, while preparations obtained from fruits (apples, lemons) and vegetables are characterized by a relatively high content of SDF. Insoluble fiber swells in the aquatic environment, filling the intestines, which regulates their peristalsis and prevents them from straining. Water-soluble fiber forms a sticky gel in its environment. This property reduces blood cholesterol, regulates glucose, reduces the absorption of sugars, and gives a feeling of satiety. Therefore, it is important to be able to influence the content of fiber fractions in the product, showing a special health-promoting impact or having a functional effect in the technological process [6,7].

Application of dietary fibers in formulation of food products depends on their functionality. Interaction with other ingredients, such as starch, is important [8]. From a functional and technological point of view, DF addition into food products contributes to the improvement and influences the rheological, textural, and sensory characteristics, as well as shelf-life of foods through its water binding capacity, gel forming ability, texturizing, and thickening effects [9,10]. The presence of non-starchy polysaccharides affects the pasting, textural, and rheological properties of starch and flour dispersions [11,12].

The rheological and textural properties affect the sensory features of food products. Therefore, knowledge of the flow characteristics and changes in the internal structure of starch pastes is extremely important at the stage of design, control, and evaluation of technological processes [13,14]. The literature presents limited information on the pasting and textural properties of starch-fiber mixtures, mainly wheat, rice, and corn starch-fiber systems [15,16,17,18,19]. However, there is limited information on the effect of some widely found fiber sources, such as oat and apple, on the rheological properties, especially thixotropic properties, of potato starch-fiber blends. For this reason, the aim of the present study was to investigate the influence of selected types of dietary fibers (fibers from oat (OF) and apple (AF)) (concentration 0.2%) on the pasting properties, rheological (including thixotropic and anti-thixotropic), and textural properties of 3% and 4% (w/w) waxy potato starch (WPS) pastes.

## 2. Materials and Methods

### 2.1. Materials

Waxy potato starch (WPS) Eliane 100, a product of AVEBE FOOD (Veendam, The Netherlands) contained 83.8% dry matter, <1% amylose, 80 mg% total phosphorus, and 0.1% lipids. Ecological dietary fiber separated from oat (OF) (90.14% dry mass (d.m.); fat 4.3 g/100 g; carbohydrates 32.4 g/100 g, protein 9.5 g/100 g) was purchased from Look Food (Lublin, Poland), manufactured in Poland. The dietary fiber separated from apple (AF) (90.16% d.m.; fat 5.73 g/100 g; carbohydrates 12 g/100 g, protein 8.39 g/100 g) was purchased from Vegamarket (Wroclaw, Poland).

### 2.2. Methods

#### 2.2.1. Pasting Characteristics

Pasting characteristics of starch (3% and 4% *w*/*w* d.m.) and mixtures of starches (2.8% and 3.8%), with an addition of the fibers from oat and apple (0.2% *w*/*w* d.m.), measuring using a Viscograph-E device (Brabender GmbH & Co. KG, Duisburg, Germany). Pasting characteristics were run from 30 to 95 °C at 1.5 °C/min rate of increase in temperature. Samples after 5 min at 95 °C were cooled to 50 °C. Constant stirring at 75 rpm was maintained.

Values measured from the pasting profile of starch-fiber blends were: T_0_ [°C], the temperature at the beginning of pasting; η_max._ (Brabender Units (BU)), the maximum viscosity; T_η max._ (°C), the temperature at the maximum of viscosity; η_95°C_ (BU), the viscosity at 95 °C; η_95°C_ after 5 min; (BU), the viscosity at 95 °C after 5 min; η_min._ (BU), the minimum viscosity; –breakdown (BD) (difference between η_max._ and η_min_.); T_η min._ (°C), the temperature at minimum viscosity; η_50°C_ (BU), the viscosity after cooling to 50 °C. Viscograph Data Correlation software was used to process the data (Brabender GmbH & Co. KG, Duisburg, Germany). All measurements were run in double.

Transformation of UnitsTorque in Brabender units (BU) may be transformed to Pascalsec or Poise by calibrating the viscograph with viscosity standard fluids.

Convert BU to centipoise (cP): 700 cmg (standard) means that 0–1000 BU equals 0–700 cmg. For a Newtonian liquid and a range of 0–700 cmg. Centipoise (cP) = BU × 3 × cmg/1000. Example: 600 BU = 420 cmg or 600 BU = 420 × 3 = 1260 cP. Convert cP to Pas: 1 cP = 1 mPas. For non-Newtonian liquids, the conversion may be slightly in error. In this paper, non-Newtonian blends were tested; therefore, the units were not converted to compare the tested properties [20].

The research was carried out by Małyszek et al. (2015) [21] and concerned assessment of factors determining accuracy in measuring rheological properties of modified starches. The authors proved that the measurements taken using the Rapid Visco Analyser (RVA) viscograph cannot be performed under a defined shear rate and they should not be expressed in absolute units (mPa·s) but in conventional Rapid Visco Analyser (RVA) units or Brabender Units (BU) units only.

#### 2.2.2. Rheological Measurements

Flow curves were carried out using an a RS6000 (Gebrueder Haake GmbH, Karlsruhe, Germany) rheometer in controlled rate of shear (CR) mode with a CC26 Ti measuring system and a 1.9 gap. Measurements were taken at a constant temperature of 25 °C. The rate of shear was raised from 0 to 300 s^−1^ for 10 min and maintained at the maximum shear rate for 1 min, then the shear rate was decreased from 300 to 0 s^−1^ over 10 min.

The flow curves up (the rate of shear was increased from 0 to 300 s^−1^ within 600 s) and down (the rate of shear was decreased from 300 to 0 s^−1^ within 600 s) were fitted to Ostwald–de Waele (1) models [22].

(1) $$\tau =K\cdot {\dot{\gamma }}^{n}$$

where *τ* is the shear stress (Pa∙s), *K* is the consistency coefficient (Pa∙s^n^), $\dot{\gamma }$ is the shear rate (s^−1^) and *n* is the flow behaviour index (-).

The areas of thixotropy and anti-thixotropy hysteresis loops (the work done to destroy and rebuild the internal structure of the sample) were calculated by summing the areas of particular trapeziums between the hysteresis loop curves “up” and “down”. The area of the single trapezium was calculated according to Sikora et al. (2015) [23] with the following formula:

(2) $$A_{T}=\frac{\left(\tau_{U\left(k-1\right)}\right)-\left(\tau_{D\left(k-1\right)}\right)+\left(\tau_{Uk}-\tau_{Dk}\right)}{2}\times {\dot{\gamma }}_{a}\left[Pa\cdot s^{-1}\right]$$

where $A_{T}$ $\left[\mathrm{Pa}\cdot s^{-1}\right]$ is the area of a single trapezium at a certain shear rate; $\tau_{U\left(k-1\right)}$, $\tau_{Uk}$ (Pa) are the shear stress values between two neighbouring measuring points on the curve down; $\tau_{D\left(k-1\right),}\tau_{Dk}$ (Pa) are the shear stress values between two neighboring measuring points on the curve up; and ${\dot{\gamma }}_{a}$ (1/s) is the average difference of shear rates between neighbouring measurement points of the curves up and down. This difference of shear rates between two curves up and down (${\dot{\gamma }}_{a}$) can be calculated as:

(3) $${\dot{\gamma }}_{a}={\dot{\gamma }}_{k}-{\dot{\gamma }}_{k-1}$$

where ${\dot{\gamma }}_{k},\;{\dot{\gamma }}_{k-1}$ are the values of higher and lower shear rate between two measuring points of the hysteresis loop (1/s).

The areas of thixotropy and anti-thixotropy were calculated as the total area of the hysteresis loop.

The graphs of the power of thixotropy (*P_T_*) (W/m^3^) in a volume of the sample (the first derivative of an oriented work executed for destruction and/or rebuilding of the inner structure of a sample over a range of time) were calculated as a function of the shear rates, according to the formula:

(4) $$P_{T}=\frac{\left(\tau_{U\left(k-1\right)}\right)-\left(\tau_{D\left(k-1\right)}\right)+\left(\tau_{Uk}-\tau_{Dk}\right)}{2}\times {\dot{\gamma }}_{p}\;\left[W\cdot m^{-3}\right]$$

where ${\dot{\gamma }}_{p}=\left({\dot{\gamma }}_{k}-{\dot{\gamma }}_{k-1}\right)/2$ (1/s) is the average value of the shear rate difference.

#### 2.2.3. Textural Properties of Gels

Samples were prepared according to Section 2.2.1, using Viscograph-E. Freshly prepared gels (30 mL) were transferred to the plastic containers. Texture measurements were performed after 24 h and 7 days of storing at 4 °C. Penetration of the samples was evaluated using the CT3 Texture Analyzer, Brookfield (Middleboro, MA, USA), equipped with Texture Pro CT V1.2 Build 9 software. The samples were compressed with a cylinder probe (ø 25.5 mm) at speed 2 mm/s, with trigger load 0.01 N and target value 10 mm. In the penetration test, the parameter of hardness was calculated as a maximum force. Proportions of starch, plant fibers and water in gelatinized samples were the same as in Section 2.2.1. The reported results were the average values of at least three replications.

#### 2.2.4. Statistics

The experimental data were calculated using Statistica v.13.3 (StatSoft, Inc., Tulsa, OK, USA). The analysis of variance was done using Duncan’s test at the confidence level of α = 0.05.

## 3. Results and Discussion

### 3.1. Pasting Characteristics

Table 1 presents the parameters of pasting characteristics of waxy potato starch (3% and 4% WPS) and the waxy potato starch-dietary fiber blends (2.8% and 3.8% WPS; 0.2% AF and OF). It can be seen that the obtained curves were typical for waxy potato starches (rich in amylopectin) presented in the literature, where the paste viscosity of WPS increased quickly and also exhibited a large breakdown [24,25,26].

An increase in the starch concentration (from 3% to 4%) caused a statistically significant decrease in the temperature of the beginning of gelatinization (T_0_) and temperature at maximum viscosity (T_η max._). On the other hand, the concentration of starch did not affect the temperature at minimum viscosity (T_η min._). Krystyjan et al. (2015) [27] also observed such a tendency, but at higher starch concentrations (4%–6%). On the other hand, the replacement of starch with fiber contributed to a statistically significant increase in the T_0_ and T_η max._ parameters in comparison with the control sample. Fiber substitution affected the temperature at minimum viscosity (T_η min._) of 3% starch pastes, reducing the value of this parameter by 5.7% in the presence of oat fiber and by 6.9% in the case of apple fiber. It should also be noted that, at a higher concentration of the mixture (4%), such a relationship was no longer observed, since the addition of both fibers did not affect the discussed parameter.

**Figure 1:**
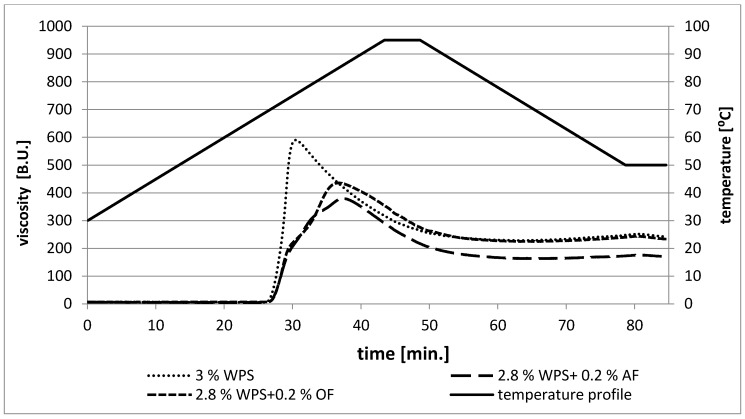
Pasting characteristics of 3% waxy potato starch and its mixtures with selected plant fibers (waxy potato starch (WPS), oat fiber (OF), apple fiber (AF)).

**Figure 2:**
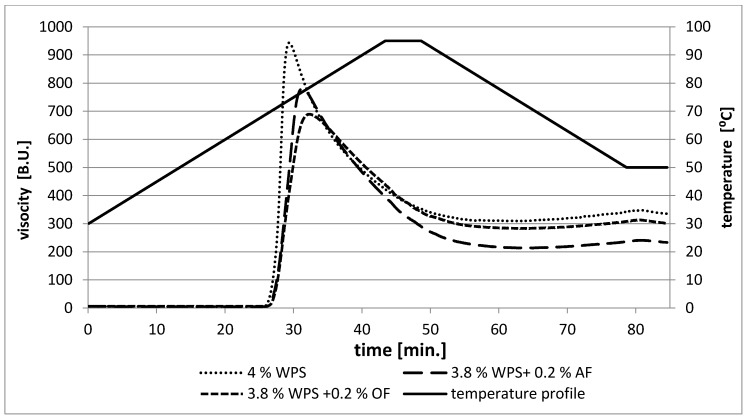
Pasting characteristics of 4% waxy potato starch and its mixtures with selected plant fibers (waxy potato starch (WPS), oat fiber (OF), apple fiber (AF)).

From Figure 1 and Figure 2, it is clear that the behavior of each blend was influenced by the type of fiber, and the effect of apple fiber was the most prominent. The peak of maximum viscosity is a measure of the degree of swelling of granules during heating. It means that starch with a higher degree of swelling gives a higher peak viscosity [28]. Maximum viscosity (η_max._) values increased with the increasing starch concentration, while the presence of fiber in starch blends caused the value of η_max._ to decrease_._ Thus, the obtained results suggest that waxy potato starch absorbed water and gelatinized at the highest range and at a lower temperature than starch-fiber blends (Figure 1 and Figure 2). Thus, the presence of fiber affects the swelling power of WPS. In samples with a total concentration of 3%, the addition of AF resulted in a greater decrease in viscosity, while, in a 4% system, the addition of OF resulted in a greater decrease in viscosity. Apple fiber particles are able to absorb water to a higher extent than starch. This could lead to less water availability in starch suspensions during gelatinization, but also to a certain compensation for viscosity loss, due to AF particle swelling and reduced leaching of amylopectin from starch granules [29,30]. In the studies by Sasaki et al. (2004) [15], the addition of fiber to starch paste caused an increase in the swelling power of wheat starch granules. The differences may result from different compositions between starches, since waxy starches contain mainly amylopectin, and, as Tester and Morrison (1990) [31] claimed, starch swelling is associated with the amylopectin behavior. According to Lai et al. (2011) [17], the content of soluble and insoluble fiber fractions influences the pasting characteristics of starches. A smaller water-soluble fraction revealed a greater swelling power. The observed significant effect on the peak viscosity values may have resulted from differences between molecular weights and macromolecular compositions (soluble and insoluble fiber contents) of studied dietary fibers [15]. Insoluble dietary fiber has a tendency toward competitive hydration due to disruption of the starch matrix during gelatinization.

The parameter of breakdown (BD) was calculated as the difference between maximum and minimum peak viscosity during heating. According to Thirathumthavorn and Charoenrein (1996) [32], a higher value of BD means less tendency of starch to resist shear force during heating and also indicates the lower stability of the final product. After gelatinization, the 3% and 4% WPS were easily broken down, resulting in the highest values of breakdown (Table 1). Based on these reports, it can be concluded that the presence of fiber in starch pastes causes a decrease in these values (BD), which may indicate better stability in starch-fiber systems than starch pastes alone. The breakdown viscosity values of the blends, including oat fibers, were lower than those of the others (WPS, WPS-AF), which suggested that they would have higher resistance to retrogradation and therefore would form a more stable paste. The same observations were described by Yildiz et al. (2013) [19] and Kaushal et al. (2012) [33] in wheat starch-oat blends.

The viscosity of starch pastes after cooling to 50 °C in relation to the minimum viscosity did not increase greatly in the tested samples. The values of viscosity at 50 °C of starch-apple and starch-oat fibers were close to the viscosity for the control sample (WPS). Apple fiber, which contains a smaller insoluble fraction than oat fiber, created less viscous systems after cooling [24,25].

#### Rheological Measurements

The flow curves, obtained in the range of increasing (0–300 s^−1^) and decreasing (0–300 s^−1^) shear rates, were described by the Ostwald–de Waele rheological model, which was characterized by a very good fit to the experimental data (R^2^ > 0.9998) (Table 2).

**Figure 3:**
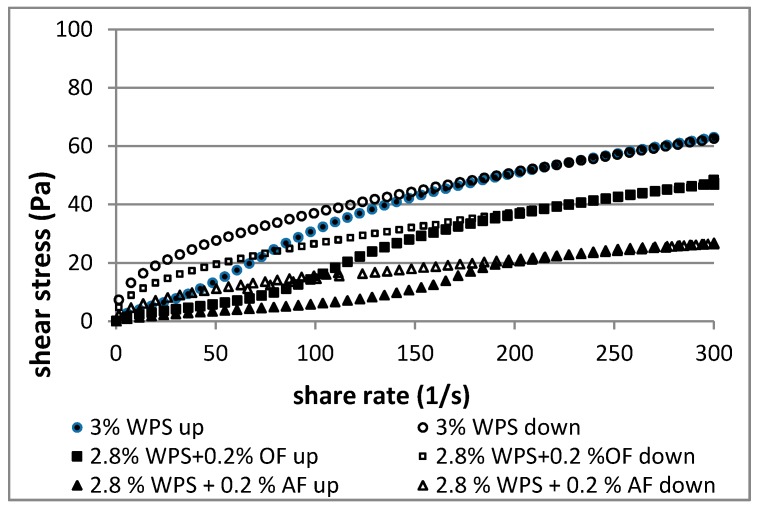
Flow curves for 3% (*w*/*w*) starch pastes and its 3% mixtures (2.8% starch with 0.2% dietary fibers) (Waxy potato starch (WPS), oat fiber (OF), apple fiber (AF)).

**Figure 4:**
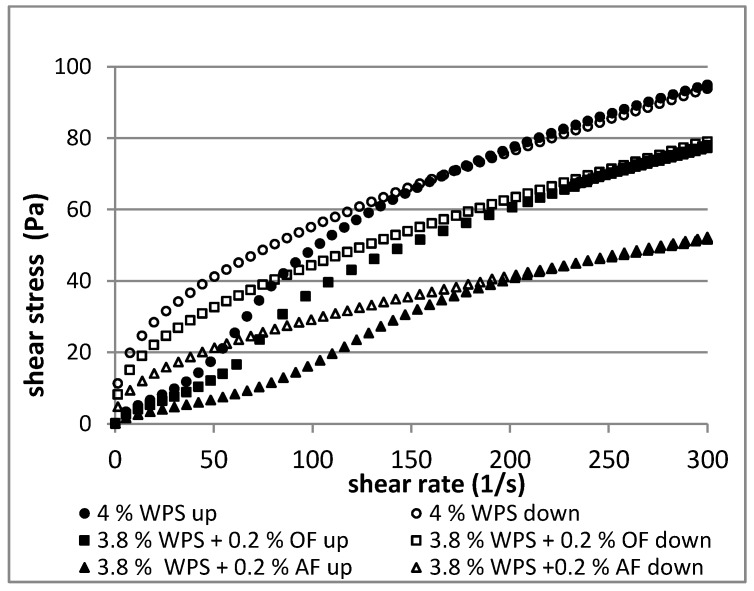
Flow curves for 4% (*w*/*w*) starch pastes and its 4% mixtures (3.8% starch with 0.2% dietary fibers) (Waxy potato starch (WPS), oat fiber (OF), apple fiber (AF)).

The pattern of flow curves (Figure 3 and Figure 4) showed that the investigated waxy potato starch and starch-fiber pastes were non-Newtonian fluids, thinned by shear. Their behavior is common for starch pastes [34,35,36].

The concentration (3%, 4%) had an influence on rheological parameters of the pastes prepared with and without addition of dietary fibers. As shown in Table 2, the consistency coefficient (*K*) and flow behavior index (*n*) of the WPS pastes increased with the concentration of WPS in the paste. The consistency coefficient (*K*) represented the shear stress values of the starch pastes during shearing, and its higher value corresponded to a higher position of the flow curves (Figure 3 and Figure 4). The 3% and 4% starch pastes exhibited pseudoplastic (shear-thinning) properties on both curves up (0–300 s^−1^) and down (300–0 s^−1^), wherein the value of the index *n* was lower in the shear range 300–0 s^–1^ than in the shear range 0–300 s^−1^. Starch pastes with substitution of AF and OF showed the higher value of index *n* than pastes without additives (WPS). The flow behavior index of starch pastes with dietary fibers was equal to or higher than one, pointing to its weak dilatation behavior in the shear range 0–300 s^−1^.

Gelatinized waxy potato starch exhibited combined hysteresis loops (Figure 3 and Figure 4), either in the direction of an anticlockwise loop (anti-thixotropy behavior) or a clockwise loop (thixotropic behavior). The anticlockwise loop direction was observed at low shear rates (0–150 s^−1^), while the clockwise loop direction was observed at higher shear rates (150–300 s^−1^), although with the first direction predominating (Table 3). Starches with a high content of amylopectin (waxy starches) exhibited the anti-thixotropy phenomenon observed as the anticlockwise direction of the hysteresis loop [37]. The anti-thixotropic hysteresis loop can be explained by the destruction of the sample structure, which occurs in the specified shear range. The formation of a new structure in the decreasing shear rate range, while the clockwise curve occurs, is a result of the destruction of the structure, i.e., thixotropic behavior [23,26,37,38,39,40].

Areas of the hysteresis loops indicated that pastes with fibers had anti-thixotropic or mixed thixotropic/anti-thixotropic character (Table 3). The starch samples containing added fiber exhibited combined hysteresis loops too, except that the 3% sample with AF (2.8% WPS + 0.2% AF) was fully anti-thixotropic. In the systems with fibers, anti-thixotropic loops dominated and thixotropic loops had smaller areas than for starch alone.

The greatest areas of the anti-thixotropy hysteresis loops were characteristic for 3% and 4% WPS (163 and 203.2 Pa/s, respectively) and its mixtures with AF (167.2 and 198.7 Pa/s, respectively). Replacing starch with oat fiber caused a statistically significant decrease in the value of these areas (46.1 and 150.1 Pa/s, respectively), which indicates that WPS–OF blends were more stable during shearing (0–300–0 s^−1^). Although oat brans absorb considerably less water than apple fiber [6], the values of total areas of the hysteresis loop were lower than in the samples with AF.

Total areas of the hysteresis loops of studied blends were smaller or not significantly different from starch alone. Oat fiber had a bigger influence than apple fiber on changing the value of the total area of the hysteresis loops. The hysteresis loop test can be used to demonstrate the rheological instability of starch pastes [38,39]. A decrease in the total area of hysteresis loops for samples with fiber may indicate rheological stabilization of the sample during its shearing.

**Figure 5:**
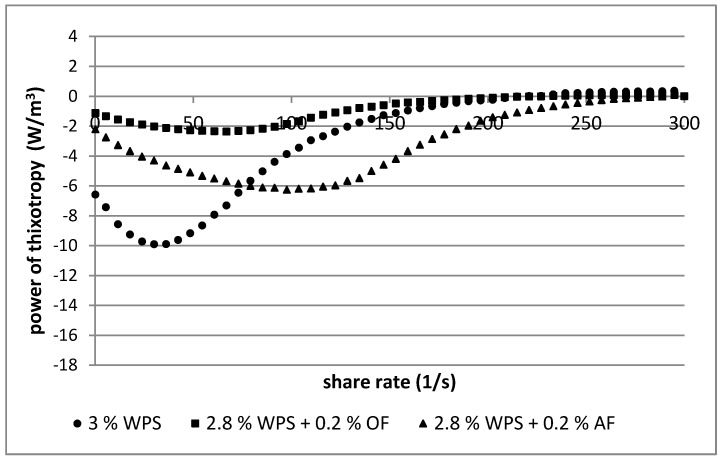
Relationship between the power of thixotropy in a volume of the sample at a defined shear rate and the shear rate in 3% (*w*/*w*) starch pastes and their 3% mixtures (2.8% starch with 0.2%dietary fibers). Waxy potato starch (WPS), oat fiber (OF), apple fiber (AF).

**Figure 6:**
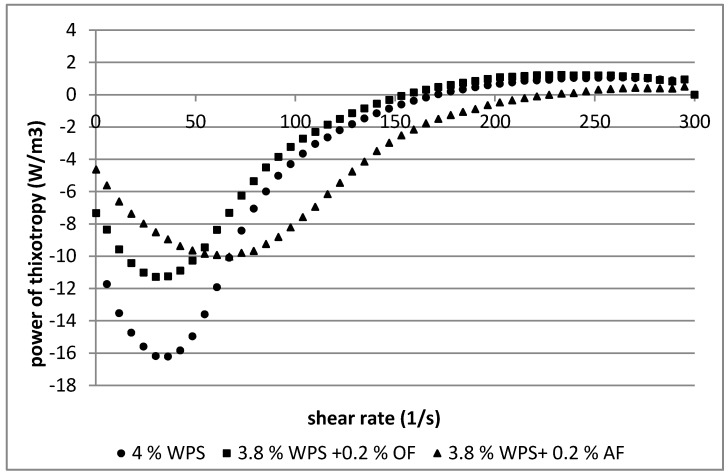
Relationship between the power of thixotropy in a volume of the sample at a defined shear rate and the shear rate in 4% (*w*/*w*) starch pastes and its 4% mixtures (3.8% starch with 0.2% dietary fibers). Waxy potato starch (WPS), oat fiber (OF), apple fiber (AF).

To determine the sample recovery rate of the original structure over the entire shear rate range, the relationship between thixotropy power as a function of shear rate was presented (Figure 5 and Figure 6) [23]. The power of thixotropy values of the samples were affected by the starch concentration (3%, 4%), the additive used (OF, AF), and the range of shear rate (0–300 s^−1^). The highest values of power of thixotropy in the pastes of starch (3%, 4%) were observed at lower shear rates (0–150 s^−1^). In this range of shear, the biggest difference between destruction and reconstruction of the internal structure of starch pastes was noticed. The same relationship occurred in starch pastes with additions of OF and AF, but to a lesser extent. In the samples with lower starch concentration (3%), addition of oat fiber had a larger impact in lowering the value of thixotropic power than apple fiber (Figure 5). At higher starch concentration (4%), an inverse relationship occurred (Figure 6).

In both starch concentrations (3%, 4%), at a lower shear rate range (0–150 s^−1^) stronger competition between destruction and recovery of inner structure of starch pastes than blends with dietary fibers was observed. Thus, in the case of starch-fiber mixtures (Figure 5 and Figure 6), in the lower shear rate range (0–150 s^−1^), less energy was needed to destroy and rebuild the internal structure.

### 3.2. Textural Properties

Table 4 shows the effect of fibers on the hardness values of starch pastes during storage.

Firstly, it was observed that replacing the starch with fibers affected the gels hardness values. Secondly, gel hardness was correspondingly the highest in samples with higher total concentration of mixtures (4%). In every case (WPS, WPS–OF, WPS–AF), the hardness of gels was higher on the 7th day of storage than after 1 day, independently of concentration of starch or type of fiber. The values of hardness for stored samples were calculated as the ratio of gel hardness after 7 days and 1 day of measurement. In the pastes with a total concentration of 3%, the largest differences in the hardness of gels during storage were observed in the starch-oat sample (2.08 of hardness increase H_7_/H_1_), while in the starch-apple pastes, this value was the lowest (1.43) (Table 4). Samples with the concentration of 4% were characterized by an inverse relationship; the highest ratio was obtained for starch-apple pastes (2.64) and the lowest for starch-oat pastes (1.82).

Fiber—type and starch concentration remarkably affected the textural parameters of the starch-fiber gels. The results can suggest that lower values of differences in hardness indicate better stability of samples in time. However, no clear trend was observed in the hardness values of the WPS–OF and WPS–AF gels. A similar result was obtained in the study of wheat and corn starch with oat and apple fibers [16,19].

## 4. Conclusions

It was found that DF can have a significant effect on WPS. As the results showed, the breakdown viscosity values of the blends, including oat fibers, were lower than those of the others (WPS, WPS–AF), which suggests that these samples would have higher resistance to retrogradation and therefore would form a more stable paste. The pasting properties of WPS with dietary fiber systems were affected by fiber replacement, as the competition for water resulted in decreased starch granular swelling, corresponding to decreased peak and final viscosities. This was confirmed by the hysteresis loop test, where the addition of oat fiber (OF) caused a statistically significant decrease in the value of the areas. Fiber type strongly affected the textural parameters of the starch-fiber gels too. The results can suggest that lower values of the gel hardness ratio after 7 days and 1 day of measurement indicate better stability of samples in time.

Furthermore, this research may be essential in the knowledge about the storage stability of DF-enriched potato starch products. The properties of the tested mixtures of starch and fiber preparations are likely affected by the content of insoluble fiber, which stabilizes such systems.

## Figures and Tables

**Table 1:** Pasting characteristics of waxy potato starch with an addition of oat and apple dietary fibers.

Samples	T_0_ [°C]	η_max._ [BU]	T_η max._ [°C]	η_95°C_ [BU]	η_95°C after 5 min_ [BU]	η_min._ [BU]	T_η min._ [°C]	BD	η_50°C_ [BU]
3% WPS	68.7 ± 0.00 ^b^	590.0 ± 0.49 ^b^	75.1 ± 0.14 ^b^	309.5 ± 2.12 ^b^	260.0 ± 1.41 ^b^	228.0 ± 2.83 ^b^	75.1 ± 0.92 ^b^	330.0 ± 7.07 ^b^	250.0 ± 2.83 ^b^
2.8% WPS + 0.2% OF	69.5 ± 0.14 ^c^	436.5 ± 23.3 ^a^	83.9 ± 0.28 ^d^	345.0 ± 8.49 ^c^	270.5 ± 4.95 ^b,c^	190.5 ± 14.85 ^a^	70.8 ± 0.28 ^a^	166.0 ± 18.38 ^a^	241.5 ± 6.36 ^b^
2.8% WPS + 0.2% AF	69.6 ± 0.07 ^c^	372.5 ± 28.9 ^a^	85.1 ± 0.85 ^d^	266.5 ± 9.19 ^a^	187.0 ± 8.50 ^a^	224.5 ± 4.95 ^b^	69.9 ± 0.28 ^a^	185.5 ± 20.51 ^a^	139.5 ± 9.19 ^a^
4% WPS	67.9 ± 0.07 ^a^	943.5 ± 55.86 ^e^	73.4 ± 0.07 ^a^	412.0 ± 15.5 ^e^	349.0 ± 14.1 ^d^	309.5 ± 10.61 ^d^	73.8 ± 0.42 ^b^	594.5 ± 41.72 ^d^	345.0 ± 12.7 ^d^
3.8% WPS + 0.2% OF	68.8 ± 0.00 ^b^	693.0 ± 21.2 ^c^	77.2 ± 0.92 ^c^	432.0 ± 2.83 ^e^	340.0 ± 0.00 ^d^	213.0 ± 4.24 ^b^	73.3 ± 1.48 ^b^	353.0 ± 21.21 ^b^	311.0 ± 4.24 ^c^
3.8% WPS + 0.2% AF	68.8 ± 0.07 ^b^	781.0 ± 11.3 ^d^	76.1 ± 0.07 ^b,c^	382.0 ± 2.83 ^d^	283.5 ± 6.36 ^c^	282.5 ± 6.36 ^c^	73.2 ± 0.28 ^b^	497.5 ± 4.95 ^c^	238.5 ± 4.95 ^b^

Parameters in columns denoted with the same letters do not differ statistically at the level of confidence α = 0.05. T_0_ [°C], the temperature at the beginning of pasting; η_max._ (Brabender Units (BU)), the maximum viscosity; T_η max._ [°C] the temperature at the maximum of viscosity; η_95°C_ (BU), viscosity at 95 °C; η_95⁰C after 5 min_ (BU), the viscosity at 95 °C after 5 min; η_min._ (BU), the minimum viscosity; –breakdown (BD) (difference between η_max._ and η_min_.); T_η min._ (°C), the temperature at minimum viscosity; η_50°C_ (BU), viscosity after cooling to 50 °C. Waxy potato starch (WPS), oat fiber (OF), apple fiber (AF). Parameters in columns denoted with the same letters do not differ statistically parameters at the level of confidence α = 0.05.

**Table 2:** Parameters of steady flow measurements of starch pastes and their mixtures with dietary plant fibers.

Sample	Ostwald–de Waele Model					
	K (Pa∙s^n^)		n (-)		R^2^	
	0–300 s^−1^	300–0 s^−1^	0–300 s^−1^	300–0 s^−1^	0–300 s^−1^	300–0 s^−1^
3% WPS	1.02 ± 0.08 ^b^	4.70 ± 0.16 ^c^	0.73 ± 0.02 ^d^	0.45 ± 0.00 ^a^	0.992	0.9999
2.8% WPS + 0.2% OF	0.19 ± 0.09 ^d^	2.87 ± 0.27 ^b^	0.99 ± 0.08 ^b^	0.49 ± 0.01 ^b^	0.988	1.0000
2.8% WPS + 0.2% AF	0.02 ± 0.00 ^e^	1.26 ± 0.25 ^a^	1.27 ± 0.02 ^a^	0.53 ± 0.02 ^c^	0.994	1.0000
4% WPS	1.55 ± 0.10 ^a^	6.94 ± 0.10 ^d^	0.73 ± 0.02 ^d^	0.45 ± 0.00 ^a^	0.988	0.9998
3.8% WPS + 0.2% OF	0.85 ± 0.03 ^c^	4.87 ± 0.19 ^c^	0.83 ± 0.01 ^c^	0.48 ± 0.01 ^b^	0.989	0.9999
3.8% WPS + 0.2% AF	0.24 ± 0.02 ^d^	3.08 ± 0.12 ^b^	1.00 ± 0.03 ^b^	0.49 ± 0.00 ^b^	0.989	0.9999

Waxy potato starch (WPS), oat fiber (OF), apple fiber (AF); consistency coefficient (*K*) (Pa∙s^n^); flow behavior index (n) (-). Parameters in columns denoted with the same letters do not differ statistically at the level of confidence α = 0.05.

**Table 3:** Areas of hysteresis loops of starch pastes and their mixtures with OF and AF.

Sample	Areas of the Hysteresis Loops (Pa/s)		
	Antithixotropy (A)	Thixotropy (T)	Total Area (A + T)
3% WPS	163.0 ^b^	3.0 ^b^	166.0 ^b^
2.8% WPS + 0.2% OF	46.1 ^a^	0.7 ^a^	46.8 ^a^
2.8% WPS + 0.2% AF	167.2 ^b^	0.0 ^a^	167.2 ^b^
4% WPS	203.2 ^c^	16.7 ^c^	219.9 ^d^
3.8% WPS + 0.2% OF	150.1 ^b^	15.8 ^c^	165.9 ^b^
3.8% WPS + 0.2% AF	198.7 ^c^	3.5 ^b^	202.2 ^d^

Waxy potato starch (WPS), oat fiber (OF), apple fiber (AF). Parameters in columns denoted with the same letters do not differ statistically significantly at the level of confidence α = 0.05.

**Table 4:** Gel hardness (N) of waxy potato starch and its mixtures with plant fiber measured after 1 day and 7 days of storage.

Samples	Hardness [N]		
	After 1 Day of Storage H_1_	After 7 Days of Storage H_7_	H_7_/H_1_
3% WPS	0.145 ± 0.01 ^a^	0.263 ± 0.04 ^a^	1.81^b^ ± 0.03 ^b^
2.8% WPS + 0.2% OF	0.180 ± 0.00 ^c^	0.375 ± 0.02 ^b^	2.08 ^d^ ± 0.02 ^c^
2.8% WPS + 0.2% AF	0.168 ± 0.01 ^b^	0.240 ± 0.01 ^a^	1.43 ^a^ ± 0.01 ^a^
4% WPS	0.223 ± 0.01 ^d^	0.430 ± 0.04 ^c^	1.93 ^c^ ± 0.04 ^c^
3.8% WPS + 0.2%OF	0.243 ± 0.01 ^e^	0.443 ± 0.03 ^c^	1.82 ^b^ ± 0.04 ^c^
3.8% WPS + 0.2% AF	0.180 ± 0.01 ^c^	0.475 ± 0.06 ^c^	2.64 ^e^ ± 0.07 ^d^

Waxy potato starch (WPS), oat fiber (OF), apple fiber (AF). Parameters in columns denoted with the same letters do not differ statistically significantly at the level of confidence α = 0.05. H_7_/H_1_—value increase expressed as the ratio of gel hardness after 7 days and 1 day of measurement.

## References

[1] Abdul-Hamid A., Luan Y.S. (2000). Functional properties of dietary fibre prepared from deffated rice bran. Food Chem..

[2] Mann J.I., Cummings J.H. (2009). Possible implications for health of the different definitions of dietary fibre. Nutr. Metab. Cardiovasc. Dis..

[3] Gallaher D.D., Schmidl M.K., Labuza T.P. (2000). Dietary fibre and its physiological effects. Essentials of Functional Foods.

[4] Lattimer J.M., Haub M.D. (2010). Effects of dietary fibre and its components on metabolic health. Nutrients.

[5] (2001). AACC Report. The definition of dietary fibre. Cereal Foods World.

[6] Chen H., Rubenthaler G.L., Leung H.K., Baranowski J.D. (1988). Chemical, Physical, and Baking Properties of Apple Fibre Compared with Wheat and Oat Bran. Cereal Chem..

[7] Korus J., Witczak M., Ziobro R., Juszczak L. (2009). The impact of resistant starch on characteristics of gluten-free dough and bread. Food Hydrocoll..

[8] Ellis R.P., Cochrane M.P., Dale M., Duffus C., Lynn A., Morrison I.M., Derek R., Prentice M., Swanston J.R., Tiller S.A. (1998). Starch production and industrial use. J. Sci. Food Agric..

[9] Thebaudin J.Y., Lefebvre A.C., Harrington M., Bourgeois C.M. (1997). Dietary fibres: Nutritional and technological interest. Trends Food Sci. Technol..

[10] Gelroth J., Ranhotra G.R., Cho S.S., Dreher M.L. (2001). Food uses of fiber. Handbook of Dietary Fibre.

[11] Biliaderis C.G., Arvanitoyannis I., Izydorczyk M.S., Prokopowich D.J. (1997). Effect of hydrocolloids on gelatinization and structure formation in concentrated waxy maize and wheat starch gels. Starch/Starke.

[12] Bonnand-Ducasse M., Della Valle G., Lefebvre J., Saulnier L. (2010). Effect of wheat dietary fibres on bread dough development and rheological properties. J. Cereal Sci..

[13] Adamczyk G., Sikora M., Krystyjan M. (2012). Methods of measuring thixotropic food products. Zywnosc-Nauka Technol. Jakosc..

[14] Adamczyk G., Krystyjan M., Dobosz A., Sikora M. (2013). Thixotropic properties of starch. Zywnosc-Nauka Technol. Jakosc..

[15] Sasaki T., Kohyama K., Yasui T. (2004). Effect of water-soluble and insoluble non-starch polysaccharides isolated from wheat flour on the rheological properties of wheat starch gel. Carboh. Polym..

[16] Cornejo-Villegas M.A., Acosta-Osorio A.A., Rojas-Molina I., Gutiérrez-Cortéz E., Quiroga M.A., Gaytán M., Herrera G., Rodríguez-García M.E. (2010). Study of the physicochemical and pasting properties of instant corn flour added with calcium and fibers from nopal powder. J. Food Eng..

[17] Lai P., Li K.Y., Lu S., Chen H.H. (2011). Physicochemical characteristics of rice starch supplemented with dietary fibre. Food Chem..

[18] Aravind N., Sissons M., Egan N., Fellows C. (2012). Effect of insoluble dietary fibre addition on technological, sensory, and structural properties of durum wheat spaghetti. Food Chem..

[19] Yildiz Ö., Yurt B., Baştürk A., Toker Ö.S., Yilmaz M.T., Karaman S., Dağlıoğlu O. (2013). Pasting properties, texture profile and stress–relaxation behaviour of wheat starch/dietary fibre systems. Food Res. Int..

[20] International Starch Institute Science Park Aarhus, Denmark ISI 19-6e Determination of Viscosity of Starch by Brabender—Comments. http://www.starch.dk/isi/methods/19brabenderNotes.htm.

[21] Małyszek Z., Makowska A., Smentek J., Kubiak P., Le Thanh-Blicharz J., Lewandowicz G. (2015). Assessment of factors determining accuracy in measuring rheological properties of modified starches. Zywnosc-Nauka Technol. Jakosc..

[22] Steffe J.F. (1996). Rheological Methods in Food Process Engineering.

[23] Sikora M., Adamczyk G., Krystyjan M., Dobosz A., Tomasik P., Berski W., Łukasiewicz M., Izak P. (2015). Thixotropic properties of normal potato starch depending on the degree of the granules pasting. Carboh. Polym..

[24] McPherson A.E., Jane J. (1999). Comparison of waxy potato starch with other root and tuber starches. Carboh. Polym..

[25] Hong Y., Zhang Y., Zhu L., Gu Z. (2011). Study on physicochemical characteristics of waxy potato starch in comparison with other waxy starches. Starch/Stärke.

[26] Krystyjan M., Sikora M., Adamczyk G., Dobosz A., Tomasik P., Berski W., Łukasiewicz M., Izak P. (2016). Thixotropic properties of waxy potato starch depending on the degree of the granules pasting. Carboh. Polym..

[27] Krystyjan M., Ciesielski W., Khachatryan G., Sikora M., Tomasik P. (2015). Structure, rheological, textural and thermal properties of potato starch—Inulin gels. LWT—Food Sci. Technol..

[28] Ragaee S., Abdel-Aal E.M. (2006). Pasting properties of starch and protein in selected cereals and quality of their food products. Food Chem..

[29] Collar C., Santos E., Rosell C.M. (2006). Significance of dietary fibre on the viscometric pattern of pasted and gelled flour-fibre blends. Cereal Chem..

[30] Goldstein A., Ashrafi L., Seetharaman K. (2010). Effects of cellulosic fibre on physical and rheological properties of starch, gluten and wheat flour. Int. J. Food Sci. Technol..

[31] Tester R.F., Morrison W.R. (1990). Swelling and Gelatinization of Cereal Starches. I. Effects of Amylopectin, Amylose, and Lipids. Cereal Chem..

[32] Thirathumthavorn D., Charoenrein S. (1996). Thermal and pasting properties of native and acid-treated starches derivatized by 1-octenyl succinic anhydride. Carboh. Polym..

[33] Kaushal P., Kumar V., Sharma H.K. (2012). Comparative study of physicochemical, functional, antinutritional and pasting properties of taro (*Colocasia esculenta*), rice (*Oryza sativa*) flour, pigeonpea (*Cajanus cajan*) flour and their blends. LWT—Food Sci. Technol..

[34] Sikora M., Kowalski S., Tomasik P. (2008). Binary hydrocolloids from starches and xanthan gum. Food Hydrocoll..

[35] Gumul D., Krystyjan M., Buksa K., Ziobro R., Zięba T. (2014). The influence of oxidation, extrusion and oxidation/extrusion on physico-chemical properties of potato starch. Starch/Starke.

[36] Krystyjan M., Ciesielski W., Gumul D., Buksa K., Ziobro R., Sikora M. (2017). Physico-chemical and rheological properties of gelatinized/freeze-dried cereal starches. Int. Agroph..

[37] Achayuthakan P., Suphantharika M. (2008). Pasting and rheological properties of waxy corn starch as affected by guar gum and xanthan gum. Carboh. Polym..

[38] Barnes H.A. (1997). Thixotropy—A review. J. Fluid Mech..

[39] Mewis J., Wagner N.J. (2009). Thixotropy. Adv. Colloid Interfaces.

[40] Wang B., Wang L., Li D., Özkan N., Li S.-J., Mao Z.-H. (2009). Rheological properties of waxy maize starch and xanthan gum mixtures in the presence of sucrose. Carboh. Polym..

